# Altered cervical posture kinematics imposed by heavy school backpack loading: A literature synopsis (2009–2019)

**DOI:** 10.4102/ajod.v10i0.687

**Published:** 2021-01-22

**Authors:** Terry J. Ellapen, Yvonne Paul, Henriëtte V. Hammill, Mariëtte Swanepoel

**Affiliations:** 1 Department of Sport and Dental Therapy, Tshwane University of Technology, Tshwane, South Africa; 2 Department of Sport, Rehabilitation and Dental Therapy, Health Science, Tshwane University of Technology, Tshwane, South Africa; 3 School of Human Movement Science, Faculty of Health Science, North-West University, Potchefstroom, South Africa

**Keywords:** cervical posture, compromised cardiopulmonary function, neuro-musculoskeletal, vertebral, proprioception, school backpack carriage

## Abstract

**Background:**

Habitual school backpack carriage causes neuro-musculoskeletal vertebral, shoulder and hand pain; deviated posture compromised cardiopulmonary function and proprioception.

**Objective:**

Present a novel literature summary of the influence of backpack carriage associated with deviated cervical posture and compromised pulmonary function.

**Method:**

An electronic literature appraisal adopting the Preferred Reporting Items for Systematic Reviews, using Google Scholar, Science Direct, EMBASE, AMED, OVID, PubMed and Sabinet search engines, was instituted during 2009–2019. Key search words: schoolbag, backpack, carriage, cervical posture and children. The quality of the studies was assessed using the Downs and Black Appraisal Scale.

**Results:**

583 records were initially identified which was reduced to 14 experimental and observational studies. A total of 1061 participants were included across the 14 studies, with an average age of 11.5 ± 1.3 years, body mass of 37.8 ± 6.6 kilograms (kg), height of 1.41 ± 0.05 meters (m), backpack mass of 5.2 ± 0.9 kg and percentage backpack mass to child’s body mass of 13.75%. The studies mean rating according to the Downs and Black Appraisal Scale was 76.3%. The average craniovertebral angle (CVA) was 53.9° ± 14.6° whilst standing without carrying a backpack was reduced to 50.4° ± 16.4° when loaded (*p* < 0.05). Backpack loads carried varied from 5% – 30% of the participant’s body mass that produced a mean CVA decline of 3.5°.

**Conclusion:**

Backpack carriage alters cervical posture, resulting in smaller CVA and compromised pulmonary function. There is no consensus of the precise backpack mass that initiates postural changes. Girls’ posture begin changes when carrying lighter backpacks as compared to boys of the same age strata.

## Introduction

Numerous investigations have been conducted in order to determine the effect of carrying school backpacks on children’s health and well-being (Dockrell, Blake & Simms 2016; Milanese & Grimmer-Somers 2010; Sharan et al. 2015). Research surveillance found that carrying a school backpack produces deviant posture, neuro-musculoskeletal and vertebral disorders (cervical and lumbar), shoulder and hand pain (Pant, Kaur & Sidhu 2016; Walikca-Cuprys et al. 2015), diminished cardiopulmonary function due to the compressive pressure of the schoolbag onto the thoracic region (Alaa & Baiee 2016; Chow et al. 2009; Veirria & Ribeiro 2014) and decreased proprioception (Mosaad & Abdel-aziem 2018) which subsequently increases the risk of falls and injuries. Subsequent research attempted to determine *safe school backpack loads*, defining weights at which negligible pain, discomfort and cervical and postural deviations were produced (Arghavani et al. 2014; Dockrell, Simms & Blake 2015; Khallaf et al. 2016). At present there is no consensus regarding what is a *safe backpack load* that produces trivial side effects in children between the ages of 10–14 years. Safe carriage loading guidelines vary from 5% to 20% relative to a child’s body mass (Dockrell et al. 2016; Hammill, Ellapen & Swanepoel 2017). The American Occupational Therapy Association recommends a load of 15% relative to the child’s body mass, whilst the American Academy of Pediatrics supports Voll and Klimt’s (1977) 10% load guideline prescription (Dockrell et al. 2016). Another factor influencing schoolbag carriage load pertains to childhood obesity and the body mass index (BMI) of the child. Overweight and obese children have larger body masses than lean children, but their muscle strength and endurance may be similar and/or even less developed (Thivel et al. 2016). In these cases, the adoption of loading guidelines, expressed as percentages (varying from 5% to 20%), may prove to be problematic as the overweight child may not have the adequate muscle strength and endurance required to carry such a load, as compared to his or her age-matched peers (Adeyemi, Rohani & Rani 2015; De Paula et al. 2012). This burden is further amplified when one takes the high prevalence of physical inactivity amongst children into account, resulting in poor musculoskeletal strength and endurance, and limited cardiopulmonary conditioning that cannot manage the load imposed by hefty school backpacks (Adeyemi et al. 2015; De Paula et al. 2012).

Hammill et al. (2017) recommended that the kinematic load carrying posture of children compared to their unloaded posture should be reviewed in order to provide biomechanical insights into determining safe loading guidelines; the present commentary is motivated by this recommendation (Hammill et al. 2017). It is a novel review of the literature pertaining to the impact of school backpack carriage on the cervical posture of children, reviewing studies published during the period 2009–2019, with specific regards to sagittal plane kinematic changes. Whilst the authors are aware that schoolbag backpack carriage influences the child’s entire vertebral column, the focus of this article is nevertheless on cervical and thoracic vertebral deviation, when viewed in the sagittal plane. Hammill et al. (2017) have already described the lower lumbar vertebrae and pelvic re-alignment induced by carrying school books, and there is therefore no need to revisit these studies. When the child carries a backpack, the weight of the load alters the incumbent’s posture in the sagittal, frontal and transverse planes because of the closed-kinetic chain interaction of all the planes (Mansfield & Neumann 2009). However, the major observable postural change is in the sagittal plane. Backpack-induced, frontal plane posture changes primarily occur when the incumbent carries the backpack on a single shoulder (unilateral carrying method) (Hammill et al. 2017). Walking is a cascade of biomechanical changes in all three planes, collectively resulting in anterior or posterior translation of the human body in the sagittal plane. Backpack-induced, static, sagittal plane postural changes can be considered as the precursory phase of the subsequent kinematic and kinetic anterior translation changes as a child walks. Therefore, this article will review backpack-induced sagittal plane postural changes amongst children, which can be used in subsequent gait kinematic research.

Although a number of tangentially related systematic reviews were completed during the aforementioned period, none of them reviewed this particular theme. Dockrell, Simms and Blake (2013) reviewed the association between prescribed backpack load guidelines and the onset of musculoskeletal pain during the period from 1984 to 2009, whilst Abdullah, McDonald and Jaberzadeh (2012) reviewed the literature related to the impact of schoolbag carriage and load placement on postural deviation amongst scholars from the 1900s until 2012. Abdullah et al. (2012) did not however review the specific changes to cervical and lumbar vertebral kinematics because of backpack loading. Hammill et al. (2017) reviewed the common anatomical sites of musculoskeletal pain induced by schoolbag carriage, methods of carrying school backpacks, the change in pelvic tilt angle and the consensus regarding the accepted safe backpack mass that can be carried by school children. However, Hammill et al. (2017) did not describe the altered cervical postural kinematics induced by carrying heavy school backpacks.

There is a paucity of literature summarising the kinematic effects of school backpack loading on cervical posture. Therefore, the authors pose the central overarching question as to whether *cervical postures change when children aged 10–14 years old carry school backpacks as compared to when they do not carry backpacks*. This central question was broken down into three more specific questions:

- What was the cascade of kinematic events, which resulted in cervical postural deviations when school children carry backpacks?
- What is the specific percent mass of backpack load that initiates changes in craniovertebral angle (CVA)?
- What is the strength of the clinical evidence supporting the ill effects of backpack loads which produce altered cervical posture amongst children?

This article presents a concise summary of the impact of heavy school backpack carriage on a child’s cervical posture by inter-relating biomechanical cascade of events occurring at the craniohorizontal angle (CHA), craniovertebral angle (CVA), shoulder sagittal angle (SSA) and anterior head alignment (AHA), which has hitherto not been undertaken. The article also reviewed the kinematic association of the altered backpack load-induced cervical posture that changes thoracic alignment, and its influence on pulmonary function. Furthermore, this is the only commentary that presents clinical evidence as per Mill’s Canons of epidemiology.

## Methods

### Protocol

An electronic, narrative literature surveillance adopting the Preferred Reporting Items for Systematic Reviews and Meta-Analyses (PRISMA) benchmarks was followed (Moher et al. 2009). The definitions were guided by the PRIMSA checklist for participants, interventions, comparisons, outcomes and study designs (PICOS) (Miller 2001). The participants were the research articles pertaining to the change in cervical posture amongst 10–14-year-old school children that carried backpack; the intervention was not necessarily a therapeutic intervention but is interpreted as an exposure, namely, the change in cervical posture of 10–14-year-old school children who carry backpacks. The outcomes of interest included (1) a cascade of kinematic events resulted in cervical postural deviations when school children carried backpacks, (2) specific percent mass of backpack loads that initiate change in craniovertebral angles and (3) gender-specific variations with regard to differing backpack mass loads relative to girls’ and boys’ body mass that can be carried without producing deviations in cervical posture.

### Participants, interventions, comparisons, outcomes and study search strategy protocol

Patient/Problem: Cervical postural changes amongst children aged 10–14 years old, who carry school backpacks.

Intervention: Change in CVA when carrying school backpacks.

Comparison: The change in children’s CVA when carrying backpacks as compared to when not carrying backpacks.

Outcome: Altered cervical posture manifested through reduced CVA, which is associated with cervical postural syndrome and thoracic kyphosis.

Research question: Does cervical posture change when children aged 10–14 years old carry school backpacks as compared to when they do not carry backpacks.

The study design of this review involved pre-test and post-test assessments

### Information sources

**FIGURE 1:**
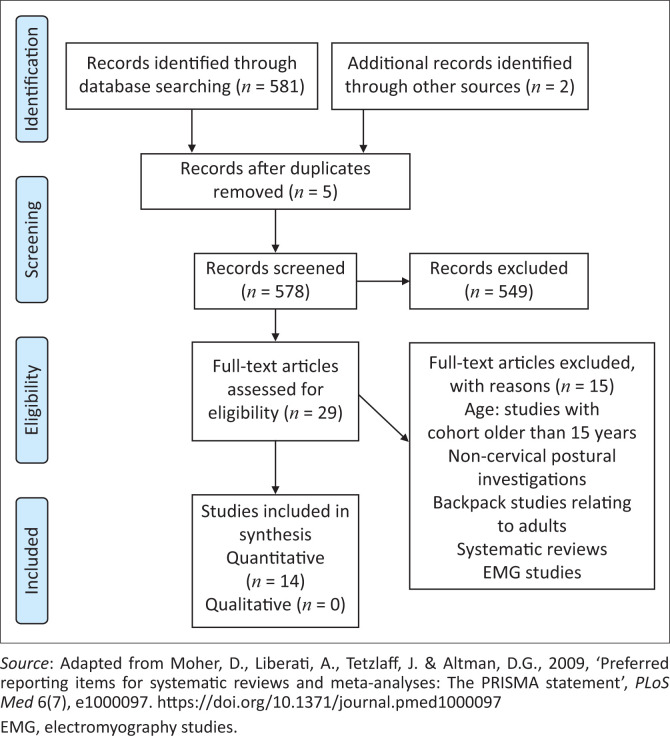
Conceptualisation of the review process.

An electronic exploration of peer-reviewed literature using the Google Scholar, Science Direct, PubMed, EMBASE, AMED, CINAHL, OVID and Sabinet search engines was completed for papers published during the period 2009–2019 (Figure 1).

### Study selection processes

The primary keyword in the literature search included ‘schoolbag carriage’; then subsequent words such as ‘backpack’, ‘cervical posture’ and ‘children’ were added. The review and selection criterion for the documents was accomplished in three phases: title review, followed by abstract review and full text review. Literature search was conducted from December 2018 until August 2019, and the records were screened by the authors (M.S., T.J.E. and H.V.H.). Each of the authors completed the three phases, resulting in a list of studies to be synthesised into the commentary. Divergent views amongst the authors whether to include or exclude a study was resolved by holding a joint review and it was put to vote whether the study should be included, based on the application of the inclusion and exclusion criteria (majority vote dictated decision).

### Inclusion criteria

Participants were records pertaining to the impact of schoolbag backpack carriage on the student’s cervical posture. Participants of the studies had to be within the age strata of 10–14 years and included both genders. The types of studies that were included in this evaluation were empirical articles and randomised control studies. Relevant themes that emerged included altered CHA, CVA and SSA because of schoolbag carriage and rehabilitative exercises were formulated to resolve the altered cervical posture of children carrying heavy schoolbags.

### Exclusion criteria

Records and articles preceding to the period prior to 2009, relating to the altered cervical posture of adults carrying backpacks, and those of children older than 15 years were not included in the study. Similarly, backpack studies relating to non-cervical posture, electromyography studies (EMG), non-English papers, meta-analyses, systematic reviews and case reports were excluded, as the authors’ primary aim was to synthesise empirical articles pertaining to the aforementioned topic.

### Quality of assessment (risk biasness)

The value of each record was assessed by adopting a modified Downs and Black Appraisal Scale, which examines the merit of randomised controlled trials and non-randomised papers (Downs & Black 1998) (Table 1). A modified Downs and Black Appraisal Scale was applied as not all of the questions on the original checklist were related to this study, as underlined by Gorber et al. (2007). These practices were employed in order to avoid any researcher bias. The modified checklist comprises 13 questions, with a maximum of 13 points. A score of either 0 (no) or 1 (yes) was given for each answer. The questions adopted from the modified Downs and Black Appraisal Scale are 1, 2, 3, 4, 6, 10, 11, 12, 13, 14, 18, 20 and 27 (Table 1). These questions are classified into four sections, which evaluate the whole merit of each record (Table 1). The classification considered the reporting prowess (*n* = 6 questions), external validity (*n* = 3 questions), internal validity (*n* = 3 questions) and power of significance (*n* = 1 question) of each publication (Downs & Blacks 1998). The reporting sub-section reviews the studies’ aims, sample characteristics and the outcome measures. The external validity reviews the representativeness of findings, and whether they can be generalised from the population the subjects were recruited from (Downs & Black 1998). The internal validity reviews whether subjects were blind to interventions used, and whether the statistical analyses were appropriate. The power of significance reviews whether the statistical tests used were adequate to determine clinically important findings (Downs & Black 1998). In the event of any disagreements amongst the authors (M.S., T.J.E. and H.V.H.) regarding the score of the selected records or articles, the authors were able to query the scoring of each record and would then discuss the scores adopting the jointly accepted score. The cumulative score of each record was subsequently converted into a percentage, thereby appraising the overall merit of the individual records (Downs & Black 1998). The overall merits of the records were further classified into the following scale: less than 50% (weak), 50% – 69% (fair), 70% – 79% (good) and less than 80% (very good) (Li, Khoo & Adnan 2017). The mean rating of the selected papers was 76.3% (good).

**TABLE 1:** The questions in the modified Downs and Black Appraisal Scale.

Question	Yes (score = 1)	No (score = 2)
**Reporting**		
1. Is the hypothesis/aim/objective clearly described?		
2. Are the main outcomes to be measured clearly described in the Introduction or Methods sections?		
3. Are the characteristics of the patients included in the study clearly described?		
4. Are the interventions of interest clearly described?		
6. Are the main findings of the study clearly described?		
10. Have the actual probability values being reported for the main outcomes, except where the probability value is less than 0.001?		
**External validity**		
11. Were the subjects asked to participate in the study representative of the entire population from which they were recruited?		
12. Were those subjects who were prepared to participate representative of the entire population from which they were recruited?		
13. Were the staff, places and facilities where the patients were treated representative of the treatment majority receive?		
**Internal validity bias**		
14. Was an attempt made to blind study subjects to the intervention they received?		
18. Were the statistical tests used to assess the main outcomes appropriate?		
20. Were the main outcome measures used accurately? (validity and reliability)		
**Power of significance**		
27. Did the study have sufficient power to detect clinically important effect where the probability value for a difference was less than 5%?		

*Source*: Adapted from Downs, S.H. & Black, N., 1998, ‘The feasibility of creating a checklist for the assessment of the methodological quality both of randomized and non-randomized studies of health care interventions’, *Journal of Epidemiology in Community Health* 52(6), 377–384. https://doi.org/10.1136/jech.52.6.377

### Data extraction

The following data were extracted from 14 articles:

- **Sample:** reported on the size, gender, age, height, body mass of participants and mass of backpack (expressed as a percent of the student’s relative body mass).
- **Research design:** studies were classified as randomised control trial, experimental group with concurrent control, and experimental group without control group.
- **Aim:** described the aims of the individual studies.
- **Protocols:** reported on how sagittal plane posture was analysed, how measurements of CVA, and in some studies CHA and SSA, were carried out.
- **Intervention:** the carrying of school backpack.
- **Findings:** reported on the change in sagittal plane posture when children carried school backpacks (loaded phase) versus not loaded phase.

### One search

The authors completed a search in the Sabinet database under the categorisation Medicine and Health. The preliminary search word used was ‘schoolbag’ that yielded 40 records. Then the subsequent word ‘schoolbag carriage’ was entered that yielded nine records. These records were then reviewed for relevance with respect to title and year of publication and it yielded two records. A similar search strategy was completed with regard to the other search engines.

### Synthesis of results

Descriptive statistical analyses including mean and percentages were performed. An inferential statistical paired T-test that compared the change in CVA during the unloaded versus loaded phases of the 14 studies was also completed (with the probability factor set at 0.05). The descriptive analyses involved calculating the sum of the participants in the 14 studies, then calculating their mean age, body mass, height and backpack mass. The backpack mass was then expressed as a percentage relative to the average body mass of the participants. The mean CVA of the participants when loaded (carrying traditional type backpacks) and unloaded (not carrying backpacks) was calculated and compared to determine changes in sagittal plane posture. The studies that employed an exercise intervention to combat the effect of heavy backpack post-test CVA results were omitted from the above calculation of the mean CVA and its subsequent comparison.

### Definitions of biomechanical terminology

In order to completely comprehend the cascade of sagittal plane kinematics (deviated cervical posture) when carrying school backpacks, a number of terms will need to be defined. The CHA is created by drawing a horizontal line bisecting the tragus of ear and another line drawn from the tragus to the external canthus of the eye (Hande et al. 2012). Hande et al. (2012) further recommended that this is an approximation of the head on neck angle, which is established in relation to the upper cervical spine. The CVA is created at the juncture between the horizontal line drawn through the spinous process of cervical vertebra seven (C7) and a subsequent line drawn to the tragus of the ear. It is an approximation of neck on cervical vertebrae and head alignment in relation to the thoracic vertebrae. A small CVA is suggestive of a forward head posture (Hande et al. 2012). The SSA is the angle created at the juncture between the horizontal line drawn through C7 and a corresponding line drawn between the mid-point of the greater tuberosity of the humerus and posterior aspect of the acromion (Hande et al. 2012). Hande et al. (2012) reported that this angle identifies a forward shoulder position with a smaller angle, suggesting that the shoulder is positioned further anterior than C7 (rounded shoulder).

### Ethical consideration

This article followed all ethical standards for research without direct contact with human or animal subjects.

## Results

### Study characteristics

The 14 studies comprised 13 experimental, observational and cross-sectional studies, without concurrent controls (Abrahams et al. 2011; Goswami, Sarkar & Mishra 2017; Hande et al. 2012; Hundekari et al. 2013; Khallaf et al. 2016; Kistner et al. 2013; Leman, Idris & Murdana 2013; Malik, Vinay & Pandey 2017; Mo et al. 2013; Mosaad & Abdel-aziem 2018; Pahwa 2013; Ramprasad, Alias & Raghuveer 2009; Vaghela et al. 2019) and one experimental study with a concurrent control (Misra, Nigm & Alagesan 2012). Whilst all studies (*n* = 14) reviewed the effects of backpack loading on cervical posture, four included interventions (Leman et al. 2013; Misra et al. 2012; Mo et al. 2013; Mosaad & Abdel-aziem 2018) (Table 2). One study reviewed the effects of exercise rehabilitation in order to resolve deviated cervical posture caused by backpack carriage (Misra et al. 2012), whilst another study compared traditional backpack loading with modified bag carriage (Leman et al. 2013). Mosaad and Abdel-aziem (2018) compared the impact of carrying a traditional style bag with that of a double-sided bag on children’s posture and proprioception. Mo et al. (2013) and Goswami et al. (2017) reported on the association between altered cervical posture, gait analyses and backpack loading. Of the 14 studies considered, eight studies were conducted in India (57.1%), two studies in Egypt (14.2%) and one in each of the following: South Africa (7.1%), United States of America (7.1%), Canada (7.1%) and Indonesia (7.1%).

**TABLE 2:** Results of individual studies (*n* = 14) pertaining to the influence of school backpack carriage on children’s cervical posture (2009–2019).

Authors/countries	Aim	Method	Findings
Ramprasad et al. (2009), India	To examine the alteration amongst postural angles associated with backpack loads of differing masses amongst preadolescents	Research design: experimental observation without concurrent control. Sample: *n* = 200 boys, age (12.5 ± 0.5 years), stature (1.42 m ± 0.07 m), body mass (30.9 kg ± 4.3 kg). Digitally recorded CVA, head on neck (HON), head and neck on trunk (HNOT) and lower limb angles were captured. These postural angles were compared with unloaded values versus values with backpacks weighing 5%, serially increasing by 5% (5% – 25%) of the child’s relative body mass.	The CVA significantly altered after backpack loads of 15% were carried (*p* < 0.05). The HON and HNOT angles significantly altered after backpack loads of 10% were carried (*p* < 0.05). Trunk and lower limb angles significantly altered after backpack loads of 5% were carried to relative the child’s body mass (*p* < 0.05).
Abrahams et al. (2011), South Africa	To investigate the prevalence of schoolbag carriage, musculoskeletal pain and the impact thereof on children’s CVA	Research design: experimental without concurrent controls Sample: *n* = 187 (boys: 84 and girls: 103), age (12.4 ± 0.6 years), stature (1.55 m ± 0.08 m), body mass (48.2 kg ± 12 kg), mass of schoolbag (5.8 kg ± 2.1 kg) Readings regarding posture were digitally captured in the sagittal plane with and without schoolbags. The CVA was measured.	Pubescent children carrying school backpacks experience musculoskeletal pain and altered sagittal posture. Unloaded CVA (33.2°) changed during the loaded phase (30.4°) (*p* < 0.0001).
Hande et al. (2012), India	To investigate the change in CHA, CVA, and shoulder posture in the sagittal plane when carrying school backpacks	Research design: experimental without concurrent control. Sample: *n* = 100 boys, age (13.2 ± 0.5 years), stature (1.41 m ± 0.1 m), body mass (30.4 kg ± 5.4 kg), backpack weight (4.9 kg ± 0.6 kg), percentage backpack weight relative to child’s body mass (16.6% ± 3.6%). Craniohorizontal and craniovertebral angles as well as shoulder posture in the sagittal plane without backpack and with school backpacks were recorded using AutoCAD 2004.	Children’s CVA and SSA were significantly reduced when carrying school backpacks, whilst CHA increased (*p* < 0.01).
Misra et al. (2012), India	To determine the success of exercise therapy in preventing postural deviation caused by carrying heavy backpack amongst school children	Research design: experimental with concurrent control Sample (*n* = 40): Age (12.1 ± 1.4 years), stature (1.45 m ± 0.08 m), body mass (38.5 kg ± 4.8 kg), backpack mass (7.1 kg ± 2.0 kg). Experimental group: Without load CVA (59.5° ± 7.9°), CHA (17.7° ± 4.5°), with load CVA (54.2° ± 8°), CHA (21.4° ± 5.3°). Control group: Without load CVA (59.4° ± 7.5°), CHA (18.9° ± 4.8°), with load CVA (55.4° ± 8.1°), CHA (21.4° ± 5.3°). The experimental group completed a structured 6-week exercise programme lasting 30 min each day, whilst control group subjects did not undertake an exercise programme.	There was a significant change in CVA and CHA amongst the experimental group (*p* < 0.05). Structured exercise programmes are successful in correcting the change in CVA and CHA caused by heavy backpack carriage amongst school children, thereby maintaining better vertebral posture.
Hundekari et al. (2013), India	To investigate the change in CHA, CVA and shoulder posture in the sagittal plane when carrying school backpacks	Research design: experimental without concurrent control. Sample size: 87 healthy school children – 40 girls and 47 boys – were divided into three groups depending on the percentage of school backpack mass relative to their body mass: Group 1: <10%, Group 2: 10% – 20% and Group 3: 20% – 30%. Craniohorizontal, craniovertebral and shoulder angles in the sagittal plane were recorded. Group 1: age (11.2 ± 1.0 years), stature (1.39 m ± 0.08 m), body mass (49.5 kg ± 10.5 kg), schoolbag mass (4.5 kg ± 0.9 kg: 9%). Group 2: age (10.2 ± 1.1 years), stature (1.33 m ± 0.06 m), body mass (37.4 kg ± 7.2 kg), schoolbag mass (5.6 kg ± 1.1 kg: 15%). Group 3: age (10.3 ± 1.0 years), stature (1.31 m ± 0.07 m), body mass (29.6 kg ± 5.8 kg), and schoolbag mass (6.8 kg ± 1.1 kg).	The CHA progressively increased as their school backpack load increased, whilst their CVA and SSA simultaneously decreased (*p* < 0.05).
Kistner et al. (2013), the United States of America	To examine the influence of carrying backpack loads (20%) of the children’s relative body mass on the posture and complaints of pain	Research design: Experimental without concurrent control The children’s craniovertebral, forward trunk lean and pelvic tilt angles were recorded in the sagittal planes from photographs of 62 children. The cohort’s mean age (9.7 ± 1.0 years), stature (1.43 m ± 0.01 m) and body mass (39.0 kg ± 9.2 kg) were recorded. Angles were measured standing and walking with backpacks weighing 10%, 15%, and 20% of the child’s relative body mass. The children’s subjective pain complaints were assessed by employing a visual analogue scale after walking.	The children’s CVA, trunk forward lean and pelvic tilt angles progressively decreased with increased backpack loads (*p* < 0.05). Craniovertebral angles decrease and this is suggestive of craniovertebral protrusion. Pain and discomfort increased when carrying backpacks.
Leman et al. (2013), Indonesia	To compare the changes within the CVA and SSA when carrying a traditional versus a modified school backpack	Research design: experimental without concurrent control. 34 boys’ CVA and SSA in the sagittal plane were recorded whilst carrying a traditional backpack and a modified backpack for 10 min. The load was 15% of their relative body mass. Cohort age (11.3 ± 0.4 years), stature (1.4 m ± 0.07 m), body mass (34 kg ± 7.3 kg) was recorded.	When the boys carried the traditional backpacks, their CVA and SSA became smaller as compared to the modified backpack (*p* < 0.05). Boys could carry the modified backpack for a longer period of time.
Mo et al. (2013), China and Canada	Determine the postural changes amongst children during planned and unplanned gait termination	Research design: Experimental, observational without concurrent control Sample: *n* = 12 boys, mean age (9.9 ± 1.3 years), stature (1.4 m ± 0.1 m) and body mass (35.0 kg ± 9.6 kg). The boys walked across an 8-m walkway at a comfortable pace carrying an unloaded backpack (0% BM), which was then loaded at 10% and 15% relative to their body mass. The boys initially stood on a force plate in an anatomical position with 0% BW backpack load in order to measure CVA, CHA, SSA, AHA and coronal shoulder posture angle (CSPA). The study involved two conditions: condition 1: unplanned gait termination and condition 2: planned gait termination. The following postural angles were digitally measured: CVA, CHA, SSA, AHA and CSPA.	Gait termination, irrespective of whether it was planned or not, did not produce remarkable postural changes. The CVA and SSA were significantly smaller during planned gait termination compared to unplanned gait termination under loaded conditions of 10% and 15% as compared to the measurements taken for the unloaded backpack (*p* < 0.05).
Pahwa (2013), India	To establish the amount of backpack mass load which, when carried by school children, does not alter their cervical and shoulder alignment	Research design: experimental observational without concurrent control. Sample: *n* = 10 boys, age (14.1 ± 1.1 years), stature (1.45 m ± 0.08 m), body mass (44.3 kg ± 7.8 kg). The boys were photographed in the sagittal plane and anterior frontal plane without backpacks and were then photographed carrying a backpack weighing from 8% to 20% of their body mass (continuously adding 1% of their relative body mass until the weight reached 20%). Four angles were electronically measured: CVA, CHA, shoulder sagittal angle (SSA) and anterior head alignment (AHA).	Cervical posture (CVA, CHA, SSA and AHA) began to change when boys carried loads at 9% of their relative body mass, a result well below the previously accepted threshold of 15% of their relative body mass. The CVA, SSH and AHA of the boys decreased when carrying schoolbags, whilst their CHA increased.
Khallaf et al. (2016), Egypt and Saudi Arabia	To examine the influence of various school backpack loads on cervical posture when standing and walking	Research design: experimental without concurrent control Sample: 100: Boys (*n* = 50): age (12.2 ± 1.6 years), stature (1.46 m ± 0.08 m), body mass (45.8 kg ± 8.2 kg); Girls (*n* = 50): age (11.9 ± 1.6 years), stature (1.45 m ± 0.09 m), body mass (45.2 kg ± 9.9 kg) Craniovertebral protrusion (sagittal plane) and lateral cervical angles (frontal plane) were measured when children carried a backpack weighing 5%, 10% and 15% of their relative body mass, both when standing and whilst walking 100 m. Decreased CVA indicates craniovertebral forward protrusion.	Girls’ craniovertebral forward protrusion increased and lateral cervical angles significantly decreased at backpack loads of 5%, 10% and 15% of their relative body mass (*p* ≤ 0.05). Boys’ craniovertebral forward protrusion increased and lateral cervical angles decreased at their backpack loads at 10% and 15% of their relative body mass (*p* ≤ 0.05). Craniovertebral forward protrusion was accompanied by decreased CVA.
Goswami et al. (2017), India	To examine the effects of school backpack load carriage and duration relating to cervical postural deviation. Subsequently normative school backpack mass loads were recommended.	Research design: experimental without concurrent control. Six male children, mean age: 10.7 ± 0.4 years, stature: 1.34 m ± 3.6 m and body mass: 29.6 kg ± 2.5 kg. Loads were 0%, 8%, 12% and 16% of their relative body mass on a treadmill moving at a speed of 1.1 m/s – 1.4 m/s for 20 min. The children’s kinematics in the sagittal plane was recorded at selected time intervals: 0, 5, 10, 15 and 20 min. The forward cervical angle (craniovertebral angle) was measured.	As schoolbag loads increased, there was an associative forward cervical inclination. Schoolbag loads weighing 12% and more of the child’s body mass significantly altered cervical posture. It is recommended that children carry school backpacks weighing no more than 8% of their body mass in order to prevent decreased CVA.
Malik et al. (2017), India	To determine the impact of carrying different backpack loads on children’s sagittal plane cervical kinematics	Research design: experimental without concurrent control Sample: 30 children (15 boys and 15 girls), age (11.9 ± 2.5 years), stature (1.38 m ± 0.07 m), body mass (49.2 kg ± 9.2 kg) and mass of school backpack (4.3 kg ± 1.2 kg). Craniovertebral angle and SSA were measured when the children carried no load, further measurements were taken with a right shoulder load, and with loads on both shoulders at 10%, 15% and 20% of their body mass.	Cervical and spinal angles changed in association with corresponding load increments. Craniovertebral angle and SSA progressive decreased as the loads increased as a result of load compensation.
Mosaad and Abdel-aziem (2018), Egypt	To compare children body’s proprioception/ balance and CVA, CHA and shoulder angles when carrying a double-sided bag as to a traditional backpack	Research design: experimental without concurrent control Sample: 33 children (19 boys and 14 girls), mean age: (9.9 ± 1.1 years), body mass (32.1 kg ± 4.3 kg), stature (1.35 m ± 0.05 m). Every child participated in three loading conditions: no load, tradition­al backpack (15%) and double-sided bag (15%). The proprioception, CVA and CHA angles were assessed in these loading conditions.	The overall and anteroposterior proprioception indices were significantly higher when loaded with the traditional backpack as com­pared to no load and to the double-sided bag (*p* < 0.05). The mediolateral proprioception index was significantly higher when carrying the traditional backpack load and the double-sided bag compared to no load (*p* < 0.05). The CHA was significantly greater, and the CVA and SSA were significantly lower when carrying the traditional back­pack as compared to no load and the double-sided bag (*p* < 0.05). It seems that carrying a double-sided bag may restore body balance and cervical posture, similar to the unloaded condition.
Vaghela et al. (2019), India	To determine the impact of backpack loading on cervical and sagittal shoulder posture (SSP) statically and after dynamically amongst school children	Research design: experimental observation without concurrent control. Sample: *n* = 160 (89 boys and 71 girls); mean age (10–15 years), body mass (34.8 kg ± 9.8 kg), mass of backpack (6.4 kg ± 1.4 kg). Twenty children were selected from each age strata from 10 to 15 years. The 160 selected children completed a questionnaire. The backpack mass carried was 18% of the child’s relative body mass over both shoulders. The following postural angles were measured: CVA, CHA and SSP.	The average CVA (40.62° ± 10.1°), CHA (20.5° ± 8.1°) and SSP (39.3° ± 4.3°) were recorded without backpacks. The average CVA (36.1° ± 10.5°), CHA (24.5° ± 10.3°) and SSP (54.3° ± 21.1°) whilst standing and carrying a backpack weighing 18% of the child’s relative body mass were then recorded. Following this the average CVA (33.8° ± 7.9°), CHA (28.9° ± 4.3°) and SSP (77.6° ± 17.5°) after dynamic activities carrying a backpack weighing 18% of the child’s relative body mass were recorded. There was a significant change in CVA, forward protrusion of the head position, CHA and SSP when carrying backpack loads weighing 18% of the child’s relative body mass.

AHA, anterior head inclination; BM, body mass; BW, body weight; CHA, craniohorizontal angles; CSPA, coronal shoulder posture angle; CVA, craniovertebral angles; HON, head on neck; HNOT, head and neck on trunk; SSA, shoulder sagittal angle; SSP, sagittal shoulder posture.

### Risk of bias assessment

The measures of the risk of bias assessment are described in Table 3. The Reporting sub-section mean was 5.07, whilst the External Validity sub-section mean was 1.85. The mean of the sub-sections of Internal Validity and Power of Significance was 2.0 and 1.0, respectively. The overall mean merit rating of the 14 records as a percentage was 76.3%, which was classified as good according to the Li et al. (2017) Scale.

**TABLE 3:** Results of the evaluation of records pertaining to schoolbag carriage cervical posture amongst students during the period of 2009–2019 (*n* = 14).

Authors	Modified Downs and Black Appraisal Scale					
	Reporting (*n* = 6)	External validity (*n* = 3)	Internal validity (*n* = 3)	Power (*n* = 1)	Total (*n* = 13)	Grading % = The accumulative score/13 × 100
Ramprasad et al. (2009)	5	2	2	1	10	76.9
Abrahams et al. (2011)	5	2	2	1	10	76.9
Hande et al. (2012)	5	2	2	1	10	76.9
Misra et al. (2012)	6	2	2	1	11	84.6
Hundekari et al. (2013)	5	2	2	1	10	76.9
Kistner et al. (2013)	5	2	2	1	10	76.9
Leman et al. (2013)	5	2	2	1	10	76.9
Mo et al. (2013)	5	2	2	1	10	76.9
Pahwa (2013)	5	1	2	1	9	69.2
Khallaf et al. (2016)	5	2	2	1	10	76.9
Goswami et al. (2017)	5	2	2	1	10	76.9
Malik et al. (2017)	5	1	2	1	9	69.2
Mosaad and Abdel-aziem (2018)	5	2	2	1	10	76.9
Vaghela et al. (2019)	5	2	2	1	10	76.9

### Data synthesis

A total of 1061 participants were recorded across the 14 studies, with an average age of 11.5 years (± 1.3), which yielded an average of 13.75% backpack mass relative to the child’s body mass. The average CVA was 53.9° ± 14.6°, whilst standing without carrying a backpack (unloaded phase) was reduced to 50.4° ± 16.4° when loaded (*p* < 0.05) (Table 4). Backpack loads carried by subjects varied from 5% to 30% of the participant’s average body mass and produced a mean CVA decline of 3.5°. The participants’ average body mass was 37.8 kilograms (kg) ± 6.6 kg, height was 1.41 metres (m) ± 0.05 m and backpack mass was 5.2 kg ± 0.9 kg.

**TABLE 4:** Comparative analyses of craniovertebral angle during unloaded versus loaded phases of the 14 studies.

Unloaded CVA	Loaded CVA	*p*
53.9° ± 14.6°	50.4° ± 16.4°	0.0006

CVA, craniovertebral angle.

### Research themes

The following themes evolved from the literature review:

- The cascade of kinematic events that result in cervical postural deviation, manifested through diminished CVA.
- The need to identify the specific percent mass of backpack load that initiates changes in CVA.
- The strength of the clinical evidence supporting the ill effects of backpack loads which produce altered cervical posture amongst children.

## Discussion

The similarity of significant changes observed in the cervical curvature when viewed in the sagittal plane was clearly established because of the uniform adoption of fundamental test to measure the CVA change. This helped to improve the validity of the findings. The primary biomechanical objective was to determine through the measurement of CVA whether the carrying school backpacks alter sagittal plane cervical posture. Empirical literature subsequently revealed that altered CVA is accompanied with altered CHA and SSA. However, a common biomechanical explanation of how these altered cervical angles combined to produce a deviated cervical posture is conspicuously missing in the literature. Individual studies have identified changes in the aforementioned sagittal plane angles, but none of them explain the phenomenon holistically. The current discussion will concentrate on the following three themes: the cascade of kinematic events that result in cervical postural deviations, specific percent mass of backpack loads that initiate change in CVA, and the strength of clinical evidence supporting the ill effects of backpack loads, resulting in altered cervical posture amongst children.

### The cascade of kinematic events that lead to cervical postural deviations

Children’s sagittal plane posture was altered when they carried backpacks weighing between 5% – 20% of their relative body mass (Hande et al. 2012; Khallaf et al. 2016; Pahwa 2013; Vaghela et al. 2019). The deviation in posture was indicated by the change in CVA, CHA, and SSA. The CVA and SSA decreased progressively as backpack loads increased, whilst the CHA increased progressively (Hundekari et al. 2013). The CVA decreased in order to maintain balance within the anterior-posterior vertebral curves. The normal anterior-posture vertebral curves include marginal cervical lordosis, thoracic kyphosis and lumbar lordosis, which are responsible for aiding the vertebral column in supporting an upright posture (Mansfield & Neumann 2014). When the child carries a backpack load which is beyond the muscular strength of the erector spinae, a forward lean away from the medial-lateral axis is adopted (Kistner et al. 2013), resulting in the forward movement of their centre of gravity (anteroposterior index) (Mosaad & Abdel-aziem 2018), thereby increasing the risk of falling forward. In an attempt to avoid falling and simultaneously securing the backpack, the child compensates by hyper-extending their lumbar vertebrae (excessive lordosis), then hyper-flexing their thoracic vertebrae (excessive kyphosis) and anteriorly protruding their cervical vertebrae (diminished CVA, resulting in cervical postural syndrome). When the child’s CVA decreases, the child’s CHA increases so as to maintain the head in an upright position, producing altered kinetic chain affects which ripple down the lower vertebrae. This kinematic vertebral change decreases CVA and SSA, but simultaneously increases CHA (Hande et al. 2012; Hundekari et al. 2013). The spontaneous re-alignment of vertebrae in order to maintain balance and an upright standing posture is known as serial distortion of the kinetic chain (Prentice 2011). Habitual carrying of heavy backpacks produces a kypholordotic posture with cervical postural syndrome (decreased CVA and SSA, coupled with increased CHA). Furthermore, the posterior kyphosis produces an anterior sunken chest (pes cavus) that may impact the child’s ventilation. Clinical literature has confirmed that carrying hefty school backpacks reduces the subject’s lung volume (Alaa & Baiee 2016; Ramadan & Ali-Shayea 2013; Veirria & Ribiero 2014). The sunken chest produces a decrease in the intra-rib spacing, bringing the superior ribs closer to the inferior ribs (Hammill et al. 2017). This action asymmetrically strengthens the internal intercostal muscles (responsible for expiration), whilst simultaneously elongating the external intercostal muscles (responsible for inspiration) (Mansfield & Neumann 2009). The asymmetrical alteration of the resting length tension relationship of these force-couple muscles produces a negative impact on the child’s inspiration, producing chronic restrictive pulmonary disorder, thereby diminishing their forced vital capacity and inspiratory lung volumes (Mansfield & Neumann 2009; McArdle, Katch & Katch 2015).

However, Misra et al. (2012) reported that specific muscle strengthening and endurance conditioning help to resolve habitual cervical postural syndrome amongst children carrying heavy backpacks. Similarly, Prentice (2011) advocated that therapeutic resistance strengthening of the thoracic erector spinae muscles can reduce the presence of kyphosis, whilst simultaneously stretching the anterior chest muscles (pectoralis major and minor, serratus anterior and the internal intercostals). The impact of the improved muscle strength, endurance and posture garnered from the exercise therapy in association with its influence on the child’s pulmonary functioning has however not being measured. It is recommended that this gap in the literature should be measured with specific investigations.

### Specific percent mass of backpack loads that initiate change in craniovertebral angles

Pahwa (2013), Khallaf et al. (2016) and Goswami et al. (2017) noted significantly decreased CVA once backpack loads exceeded 9%, 10% and 12% of the boys’ relative body mass, respectively. The mean age of the cohort in these studies was 10.7–14.1 years, referring to a cervical postural, change-specific age strata of 10–14 years. These findings suggest that a critical safe backpack load might be set at 8% relative to boys’ body mass for boys aged 10–14 years (if one adopts the lower percent load relative to the child’s body mass). The critical limitations of these studies were the small sample size: Pahwa (2013) (*n* = 10 boys), Khallaf et al. (2016) (*n* = 50 boys) and Goswami et al. (2017) (*n* = 6 boys). A larger sample is needed in order to validate their findings. The empirical evidence indicates that CVA changes occur when a child carries a backpack, but they differ in opinions as to what percent mass of the backpack load produces a significant CVA change. Chansirinukor et al. (2001) reported that backpack loads from 15% produce significant CVA changes, whilst Pahwa (2013) reported that loads exceeding 8% produce altered CVA. Pahwa (2013) concurs with Ramprasad et al.’s (2009) findings. These findings are conflicting in their precise percentage load value. Therefore, the authors recommend that further empirical investigations should be conducted to determine the precise percentage of backpack load that produces a significantly altered CVA, CHA and SSA, which are also associated with neuro-musculoskeletal discomfort and pain.

Gender variations were also identified. Khallaf et al. (2016) reported that girls’ CVA started to change with loads of 5% relative to their body mass. They suggest that girls’ cervical posture begins to change earlier in order to accommodate carrying heavy backpacks well before the prescribed guidelines adopted by the American Occupational Therapy Association (15%) and the American Academy of Paediatrics (10%). Khallaf et al.’s (2016) findings concur with Hammill et al.’s (2017) recommendation that boys and girls should have different *safe mass loads per age strata* in so far as their muscle strength and endurance differ. Boys and girls during their anatomical and physiological development possess different muscle strength and endurance capacities, which will impact their ability to carry different relative, percent body mass backpack loads, as well as being able to maintain anatomically correct and neuro-musculoskeletal discomfort-free, and/or pain-free posture. Males are generally stronger than females within their age-specific strata (Hammill et al. 2017; Khallaf et al. 2016) that may allow them to carry relatively greater/heavier backpack loads in relation to their body mass and adopting pain-free postures. This aforementioned evidence warrants the international paediatric health associations to prescribe *independent gender-specific safe backpack mass loads* for boys and girls.

### Strength of evidence supporting the ill effects of backpack loads producing altered cervical posture amongst children

It is a common practice to adopt Mill’s Canons (Dishman, Heath & Lee 2013) to determine the vigour of the evidence supporting casual inferences. As such, the authors embraced Mill’s Canons in order to establish the strength of evidence supporting the causal inference that carrying heavy backpacks produces cervical posture deviation amongst children:

- Temporal sequence refers to the order of exposure of the intervention, which must precede the change of the diseased condition (deviated cervical postural) within a sufficient time frame to make a plausible conclusion. A total of 11 studies reported a change in children’s cervical posture once backpacks were carried (refer to Table 4).
- Strength of association refers to the clinical significance between the disease (deviated cervical posture) and the intervention (carrying backpacks). Eleven studies indicated a strong association between deviated cervical postures when carrying backpacks (Table 4), where the intervention is regarded as the carrying of the school backpack and posture is regarded as the dependant variable.
- Consistency of results refers to the consistent observation of the association between the consequence of the intervention (carrying backpacks) and the disease (deviated cervical posture). The 11 empirical studies reported changes in children’s cervical posture when they carried backpacks (Table 4). The aforementioned studies indicated a decrease in CVA and SSA and a concurrent increase in CHA.
- Biological plausibility refers to the clinical explanation of the observed outcome of the intervention regarding diseases. The 11 studies also reported altered CVA, CHA, and SSA, indicating altered cervical posture because of carrying backpacks (Table 4). Studies have confirmed that habitual backpack loading compromises pulmonary functioning.
- Dose response refers to the volume of intervention required to produce a specific outcome on the disease. Evidence indicates that a backpack mass greater than 8% for boys and 4% for girls within the 10–14 years age group produces altered cervical posture.

## Limitations

This review was not registered with the The International Prospective Register of Systematic Reviews (PROSPERO) website. The review has identified that heavy schoolbag backpack loads alter the sagittal plane cervical posture, reflected by a diminished CVA. However, this altered cervical posture also impacts the SSA and CHA. There were only four studies that measured SSA and CHA, which reflected altered cervical posture. More investigations need to be conducted to document the changes in these associated kinematic angles. Although literature has identified gender variations relating to the extent of percent backpack mass loads that boys and girls can carry, more investigations are needed to guide international paediatric health associations to draft specific independent gender-specific safe backpack mass loads for boys and girls.

## Conclusion

Children carrying backpacks experience a change in their cervical posture, which might alter their normal day-to-day living and wellness. The child’s CVA and SSA diminish, whilst their CHA increases, thus altering the anterior-posterior curvature of the vertebrae, producing a kypholordotic posture and cervical postural syndrome. The empirical evidence indicates that CVA, CHA and SSA changes occur when a child carries a backpack but they differ in opinions as to what relative percent mass of the backpack loads produce significant CVA, CHA and SSA changes. The relatively precise percent backpack load that produces altered CVA, CHA and SSA associated with neuro-musculoskeletal discomfort and/or pain needs to be identified. It is well established that boys are usually stronger than girls within their age-specific strata, which enables them to carry relatively greater backpack loads in relation to their body mass. The altered cervical posture also poses a threat to the child ventilation. Parents, educators and healthcare professionals should consider the aforementioned literature that limits the percent mass load when children carry backpacks. This aforementioned evidence warrants the international paediatric health associations to prescribe *independent gender-specific safe backpack mass loads* for boys and girls.
